# The Choice of Operation for the Removal of Large Stones from the Bladder

**Published:** 1900-10-13

**Authors:** 


### THE CHOICE OF OPERATION FOR THE REMOVAL OF LARGE STONES FROM THE BLADDER.

In a recent discussion at Ipswich as to the best treatment of large vesical calculi, Mr. Cadge, of Norwich, made some interesting remarks in regard to the choice of operation in such cases, and in so doing he drew a broad distinction between the proceedings which might properly be undertaken by specialists who had had the advantage of considerable practical experience in operating in such cases, and general hospital surgeons who only treated these cases occasionally, saying that while the expert in stone practice might be trusted to do what he thought best, what might seem best in his experienced hands might not be best in the hands of the general surgeon. He said that at the present day the question as to the treatment of large stones seemed to lie between suprapubic cystotomy and perineal lithotrity, or rather litholapaxy, and that while to the specialist the latter might be the best, to the ordinary surgeon the former was the preferable operation. It is, he says, easier of execution than any form of perineal operation; the parts dealt with are under the eye; there are no vessels likely to be met with which will give trouble; the bladder wall to be cut can be incised to any extent, and stitched up after the stone's removal if necessary; moreover, an enlarged prostate which so often impedes perineal operations causes no difficulty, or very slight, in the suprapubic method; while, should a stone be fixed in the end of the ureter, or in some form of sac, it can be dealt with far more easily than by any other opening.

Several surgeons, however, who had devoted considerable attention to the subject, differed very strongly from the recommendations made by Mr. Cadge, and Mr. Freyer was particularly scornful. But we may be allowed to doubt whether the statistical argument which was especially relied upon by some can properly decide the question, and it must also be pointed out that the problem discussed by Mr. Cadge was not Which is the best operation? but, Which is the best for a man to perform who, from the circumstances, can only have a comparatively small operative experience in such cases? The really good point made against the suprapubic operation was that mentioned by Mr. Reginald Harrison, who showed that where there is a tendency to the formation of stone, calculous material may be deposited again and again upon the scar left by the high operation. This is a real objection, and one of great interest, and if it should be found to be operative in many cases it would form a great bar to the utility of the suprapubic cystotomy.

We cannot hide from ourselves that the whole question is one of considerable interest quite outside the mere surgical problem involved. It seems now to be definitely maintained by certain specialists that success in operating for stone depends on the use of special instruments—which of course are open to anyone—and on the possession of special experience which can only be obtained by few. This raises an ethical problem which it would require some hardihood to discuss in any medical society—where the majority of surgeons present could probably count upon their fingers the stones on which they had operated—namely, Should the general hospital surgeon undertake such cases any more than he would undertake operations, say, for cataract? The mere hinting of such a question may seem to some to savour of specialism gone mad. But it is a thing which is in the air about many operations, and will sooner or later have to be seriously discussed.

